# Clinical characteristics and disease progression of retinitis pigmentosa associated with *PDE6B* mutations in Korean patients

**DOI:** 10.1038/s41598-020-75902-z

**Published:** 2020-11-11

**Authors:** You Na Kim, Joon Seon Song, Seak Hee Oh, Yoon Jeon Kim, Young Hee Yoon, Eul-Ju Seo, Chang Ahn Seol, Sae-Mi Lee, Jong-Moon Choi, Go Hun Seo, Changwon Keum, Beom Hee Lee, Joo Yong Lee

**Affiliations:** 1 Department of Ophthalmology, Asan Medical Center, University of Ulsan College of Medicine, 88, Olympic-ro 43-gil, Songpa-gu, Seoul, 05505 Republic of Korea; 2 Department of Pathology, Asan Medical Center, University of Ulsan College of Medicine, Seoul, Republic of Korea; 3 Department of Pediatrics, Asan Medical Center, University of Ulsan College of Medicine, Seoul, Republic of Korea; 4 Department of Laboratory Medicine, Asan Medical Center, University of Ulsan College of Medicine, Seoul, Republic of Korea; 5 GCLabs, GCGenome, Yongin, Republic of Korea; 6 3billion Inc., Seoul, Republic of Korea; 7 Medical Genetics Center, Asan Medical Center Children’s Hospital, University of Ulsan College of Medicine, Seoul, Korea

**Keywords:** Genetics, Diseases

## Abstract

Due to the genotype–phenotype heterogeneity in retinitis pigmentosa (RP), molecular diagnoses and prediction of disease progression is difficult. This study aimed to report ocular and genetic data from Korean patients with *PDE6B*-associated RP (*PDE6B*-RP), and establish genotype–phenotype correlations to predict the clinical course. We retrospectively reviewed targeted next-generation sequencing or whole exome sequencing data for 305 patients with RP, and identified *PDE6B*-RP in 15 patients (median age, 40.0 years). Amongst these patients, ten previously reported *PDE6B* variants (c.1280G > A, c.1488del, c.1547T > C, c.1604T > A, c.1669C > T, c.1712C > T, c.2395C > T, c.2492C > T, c.592G > A, and c.815G > A) and one novel variant (c.712del) were identified. Thirteen patients (86.7%) experienced night blindness as the first symptom at a median age of 10.0 years. Median age at diagnosis was 21.0 years and median visual acuity (VA) was 0.20 LogMAR at the time of genetic analysis. Nonlinear mixed models were developed and analysis revealed that VA exponentially decreased over time, while optical coherence tomography parameters linearly decreased, and this was related with visual field constriction. A high proportion of patients with the c.1669C > T variant (7/9, 77.8%) had cystoid macular edema; despite this, patients with this variant did not show a higher rate of functional or structural progression. This study will help clinicians predict functional and structural progression in patients with *PDE6B*-RP.

## Introduction

Retinitis pigmentosa (RP) is one of the most common hereditary retinal diseases; it is characterized by progressive pigmentary retinal degeneration which often leads to legal blindness. Over one million patients throughout the world are affected by RP, corresponding to approximately one in 4,000 individuals^1^. To date, more than 80 genes have been associated with non-syndromic RP, and more than 35 of these genes have been associated with an autosomal recessive mode of inheritance^2^.

Among patients with autosomal recessive RP, 5–8% are known to have defects in rod-specific cyclic guanosine monophosphate (cGMP) phosphodiesterase 6β subunits (PDE6_β_)^3,4^. In the phototransduction cascade, rhodopsin becomes photoexcited after absorbing photons, and then activates transducin. An activated subunit of transducin, in turn, activates rod phosphodiesterase (PDE) by releasing the regulatory PDEγ subunit. The catalytic PDE6α and β subunits are then activated to hydrolyze cGMP; the resultant low level of cGMP leads to the closure of ion channels and therefore membrane hyperpolarization^5^. The *PDE6B* gene on chromosome 4p16.3 encodes the PDE6β subunit, and variants in this gene are associated with RP^6^. In animal models, mutations in the *PDE6B* homolog (RefSeq accession number, NM_000283.3) have also been reported to cause rod and cone degeneration^7,8^.

Due to the genotype–phenotype heterogeneity in RP and the complexities of the interactions between gene expression and environmental factors, the molecular diagnosis of this condition is very complicated^9^. Recent advances in sequencing methodologies, such as targeted next-generation sequencing (TGS) and whole exome sequencing (WES), mean that it is now feasible to conduct molecular analysis for RP in clinical practice^10^. Here, we describe the clinical and molecular characteristics of RP patients with *PDE6B* variants identified by TGS and WES.

## Results

### Demographics

A total of 15 patients were recruited from 14 Korean families; seven of these 15 patients were males (7/15, 46.7%). The baseline characteristics of patients at the time of genetic testing are presented in Table 1. The median age at examination was 40.0 years (range, 13–67). The mean follow-up duration was 3.1 years (range, 1–12). According to their clinical history, 13 of 15 patients (86.7%) experienced night blindness as their first ocular symptom at a median age of 10.0 years (range, 5–47 years) and 9 of 15 patients (60.0%) were less than 10 years old at symptom onset. Two patients experienced dyschromatopsia and decreased best-corrected visual acuity (BCVA) as their first ocular symptom at an age of 10 and 4 years, respectively. Patients were diagnosed with RP at a median age of 21.0 years (range, 11–60 years).

**Table 1:** Demographic and clinical characteristics of 15 patients from 14 families with retinitis pigmentosa associated with *PDE6B* variants; visual and retinal parameters were assessed at the time of genetic testing.

Family no.	Subject no.	Age, years	Sex	Follow-up duration, years	BCVA, LogMAR		First symptom		Age at diagnosis, years	Family history	Visual field	OCT parameters				
					OD	OS	Age at onset, years	Chief complaint				IS/OS, μm	CRT, μm	ONLT, μm	ERM	CME
1	1-III-1	14	M	3	0.00	0.00	7	Night blindness	11	No	PRS	3654	253	70	No	Yes
2	2-III-2	26	F	2	0.40	0.15	10	Dyschromatopsia	11	No	PRS	2420	248	90	Yes	Yes
3	3-III-1	13	F	1	0.40	0.20	4	Decreased VA	12	No	Center < 20°	1719	306	94	No	Yes
4	4-II-3	67	M	2	3.00	0.50	10	Night blindness	20	Yes	TVFD (OD)	473	315	21	Yes	No^a^
											PP (OS)					
5	5-III-1	17	F	1	0.15	0.00	15	Night blindness	16	Yes	center < 30°	4658	261	97	No	No
5	5-III-2	15	M	1	0.10	0.10	14	Night blindness	14	Yes	center < 30°	3889	273	89	No	No
6	6-II-3	40	F	4	0.00	0.00	30	Night blindness	40	No	N/A	2644	271	62	No	No
7	7-II-5	55	M	1	0.40	0.40	47	Night blindness	55	No	PRS	1495	235	62	Yes	Yes
8	8-II-2	33	F	1	0.00	0.40	14	Night blindness	33	Yes	PRS	4046	284	72	No	Yes
9	9-II-3	62	M	1	3.00	0.50	10	Night blindness	20	Yes	TVFD (OD)	328	195	N/A	Yes	No^a^
											PP (OS)					
10	10-II-3	50	M	3	3.00	3.00	5	Night blindness	24	No	PP	394	217	N/A	Yes	No^a^
11	11-II-5	38	M	6	0.40	0.40	10	Night blindness	21	Yes	Center < 15°	1401	230	31	Yes	Yes
12	12-II-1	44	F	12	0.40	1.00	7	Night blindness	31	No	Center < 10°	812	189	38	Yes	No
13	13-II-5	67	F	6	0.10	0.10	42	Night blindness	60	No	Center < 10°	4715	252	56	Yes	No
14	14-II-5	56	F	2	1.30	0.40	6	Night blindness	44	No	PP	803	257	35	No	Yes^a^
Median		40.0		2.0	0.40	0.40	10.0		21.0			1719.0	253.0	62.0		
Mean		39.8		3.1	0.84	0.48	15.4		27.5			2230.1	252.4	62.2		

*BCVA* best-corrected visual acuity; *LogMAR* logarithm of the minimum angle of resolution; *OCT* optical coherence tomography; *IS/OS* width of the inner segment/outer segment band; *CRT* central retinal thickness; *ONLT* outer nuclear layer thickness; *ERM* epiretinal membrane; *CME* cystoid macular edema; *PRS* paracentral ring scotoma; *TVFD* total visual field defect; *PP* pinpoint visual field; *N/A* not available; *VA* visual acuity; *OD* oculus dexter; *OS* oculus sinister.

^a^The patients who were excluded from calculating the incidence of cystoid macular edema due to diffuse foveal atrophy accompanied by residual visual field less than five degree.

**Figure 1:**
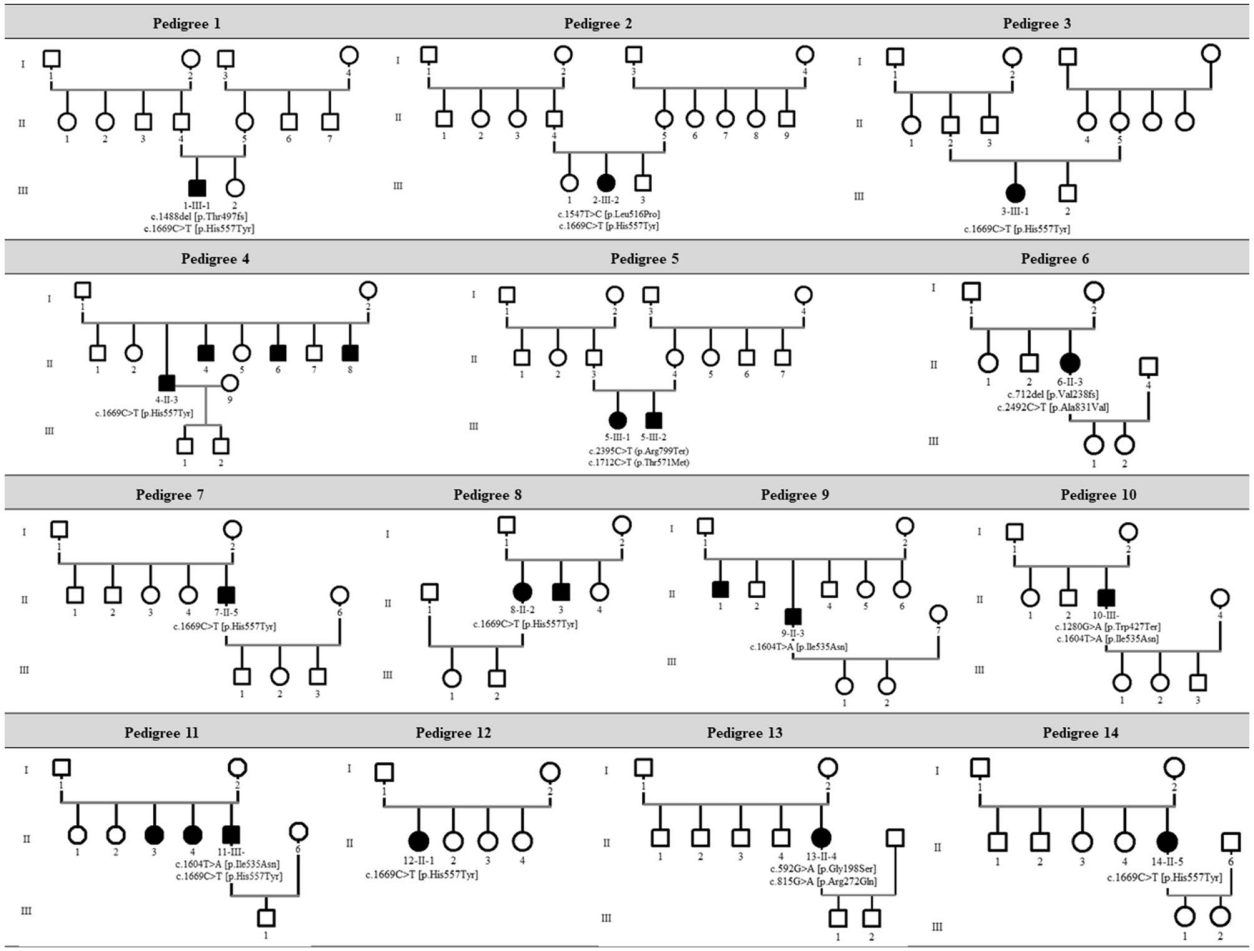
Pedigrees of 15 Korean patients from 14 families who had retinitis pigmentosa associated with *PDE6B* variants.

Five families (5/14, 35.7%) were identified to have a history of autosomal recessive RP; their pedigrees are shown in Fig. 1. Among these five families, only two siblings from one family (Pedigree 5; 5-III-1, 5-III-2) underwent segregation analysis using TGS.

### Ophthalmologic findings

**Figure 2:**
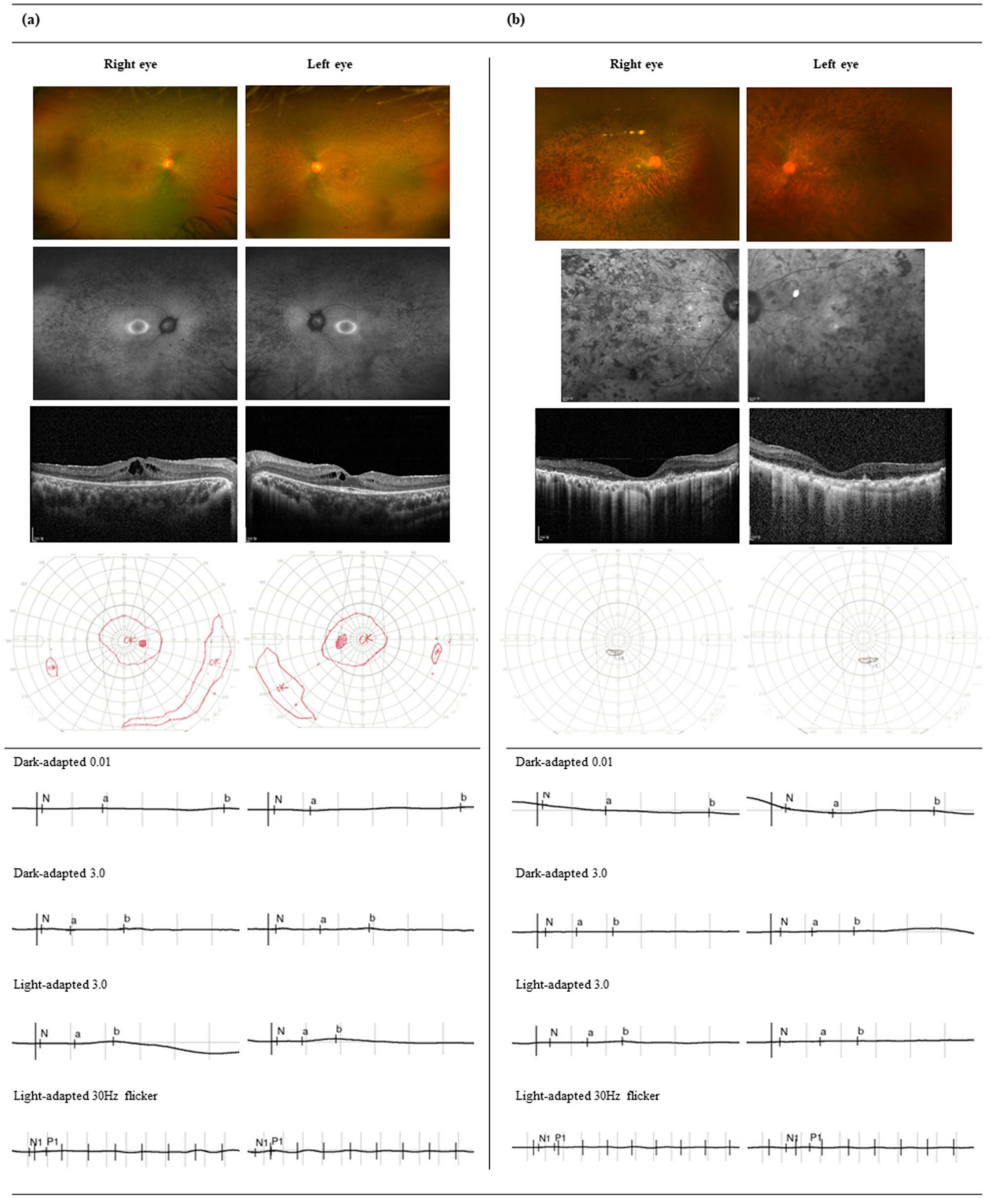
Typical ophthalmologic findings in patients with retinitis pigmentosa. From top to bottom: fundus photography images, fundus autofluorescence images, optical coherence tomography images, Goldmann kinetic visual field test results, and electroretinograms (from top to bottom) are shown for two patients, (**a**) Subject No. 2-III-2, a young patient with cystoid macular edema, and (**b**) Subject No. 9-II-3, a patient with advanced disease associated with diffuse foveal atrophy.

**Figure 3:**
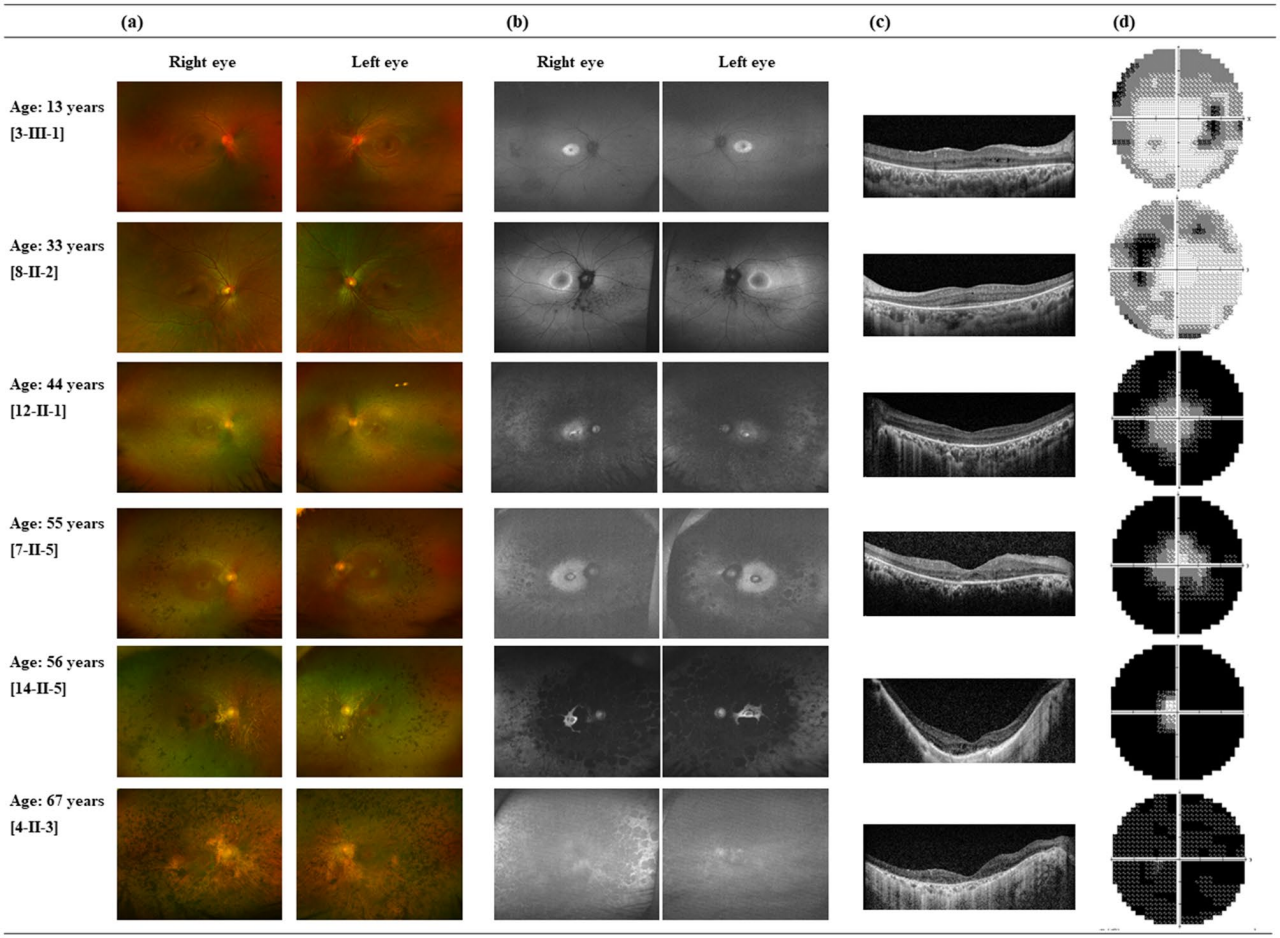
Fundus autofluorescence and optical coherence tomography images from patients homozygous for the c.1669C > T missense variant; images are ordered based on patient age. (**a**) Color fundus photography images showing the progression of pigmentary changes with age, from sparse mid-peripheral pigmentation in younger patients to coarse pigmentation invading the macula in older patients. (**b**) Fundus autofluorescence images showing a bull’s eye pattern of autofluorescence. (**c**) On optical coherence tomography images, perifoveal retinal pigment epithelium atrophy with a spared foveal anatomy and cystoid macular edema is apparent in younger patients. Diffuse neurosensory and retinal pigment epithelium atrophy involving the fovea can be observed in older patients. (**d**) Progressive visual field constriction with aging.

Ophthalmologic findings are presented in Fig. 2. Funduscopy revealed symmetrical changes typical of RP; these included fine bone spicule pigmentation in the mid-peripheral retina in all patients, and waxy pallor of the optic disc and attenuated retinal vessels in older patients. Fundus autofluorescence images revealed a bull’s eye pattern of autofluorescence with central hypoautofluorescence surrounded by macular hyperautofluorescence. The pattern of retinal pigmentation also appeared to vary with age (Figs. 2 and 3); in younger patients, funduscopy and fundus autofluorescence images showed sparse mid-peripheral pigmentation, whereas coarse pigmentation invading the macula was seen in older patients.

Examination of optical coherence tomography (OCT) images revealed that young patients showed perifoveal retinal pigment epithelium atrophy with a relatively spared foveal anatomy (Fig. 2a). In patients older than 40 years of age, OCT images revealed diffuse outer nuclear layer (ONL) and retinal pigment epithelium atrophy involving the fovea (Fig. 2b). Regarding OCT parameters, the mean width of the inner segment/outer segment (IS/OS) band was significantly lower in the eight patients over 40 years old than in the seven under 40 years old (3112.4 ± 1258.8 μm vs. 1458.0 ± 1523.2 μm, respectively, *P* = 0.041) and ONL thinning was also more pronounced in these patients (77.6 ± 23.1 μm vs. 44.3 ± 15.6 μm, respectively, *P* = 0.012). Central retinal thickness (CRT) was similar between the two age groups (265.0 ± 25.1 μm in older vs. 241.4 ± 41.7 μm in younger patients, *P* = 0.215). The presence of epiretinal membranes (ERMs) and cystoid macular edema (CME) was also assessed using OCT images (Table 1). An ERM was observed in 8 of 15 patients (53.3%); most of these (6/8, 75.0%) were over 40 years old. In terms of CME, seven patients (46.7%) had intraretinal hyporeflective cysts. The edema was limited to the inner nuclear layer (INL) in six of these patients, while only one patient (8-II-2) had CME extending from the INL to the ONL. Considering that no viable foveal tissue remains in advanced RP, we excluded the four subjects (all older than 50 years) who had diffuse foveal atrophy and a remaining visual field (VF) of less than five degrees (pinpoint VF or less). The proportion of patients with CME was then recalculated as 54.5%.

VF tests were conducted in 14 of 15 patients. Ten of these (71.4%) had a remaining bilateral central field of more than ten degrees, whereas four patients showed a severely constricted VF, defined as pinpoint or less. On full-field electroretinography, all of the patients showed extinguished or extremely low amplitude responses.

### PDE6B variants analysis

Data regarding the variants identified in the study patients are shown in Table 2. A total of 11 *PDE6B* variants were identified. Ten had been previously reported, while one was a novel variant (c.712del). Variants were classified as pathogenic (four), likely to be pathogenic (five), or of unknown significance (VUSs, two) according to the guidelines of the American College of Medical Genetics and Genomics^11^. Seven missense variants (c.1547T > C[p.Leu516Pro], c.1604T > A[p.Ile535Asn], c.1669C > T[p.His557Tyr], c.1712C > T[p.Thr571Met], c.2492C > T[p.Ala831Val], c.592G > A[p.Gly198Ser], and c.815G > A[p.Arg272Glu]), two nonsense variants (c.1280G > A[p.Trp427Ter] and c.2395C > T[p.Arg799Ter]), and two frameshift variants (c.1488del[p.Thr497fs] and c.712del[p.Val238fs]) were identified.

**Table 2:** *PDE6B* variants causative of retinitis pigmentosa in 15 patients from 14 families.

Family no	Subject no	HGVS DNA change	HGVS protein change	Zygosity	Variant type	ACMG criteria		REVEL score	Bayesian posterior probability	Primary screening method
1	1-III-1	c.1488delC	p.Thr497ProfsTer78	Hetero	Frameshift	PV	PVS1,PM2,PP5,PP4		0.999	TGS
		c.1669C > T	p.His557Tyr	Hetero	Missense	LPV	PM1,PM2,PP3,PP4,PP5	0.991	0.949	
2	2-III-2	c.1547 T > C	p.Leu516Pro	Hetero	Missense	LPV	PM1,PM2,PP3,PP4,PP5	0.900	0.949	TGS
		c.1669C > T	p.His557Tyr	Hetero	Missense	LPV	PM1,PM2,PP3,PP4,PP5	0.991	0.949	
3	3-III-1	c.1669C > T	p.His557Tyr	Homo	Missense	LPV	PM1,PM2,PP3,PP4,PP5	0.991	0.949	TGS
4	4-II-3	c.1669C > T	p.His557Tyr	Homo	Missense	LPV	PM1,PM2,PP3,PP4,PP5	0.991	0.949	TGS
5	5-III-1	c.2395C > T	p.Arg799Ter	Hetero	Nonsense	PV	PVS1,PM2,,PP4,PP5		0.999	TGS
		c.1712C > T	p.Thr571Met	Hetero	Missense	LPV	PM1,PM2,PP3,PP4	0.896	0.899	
5	5-III-2	c.2395C > T	p.Arg799Ter	Hetero	Nonsense	PV	PVS1,PM2,,PP4,PP5		0.999	TGS
		c.1712C > T	p.Thr571Met	Hetero	Missense	LPV	PM1,PM2,PP3,PP4	0.896	0.899	
6	6-II-3	c.712delG	p.Val238CysfsTer13	Hetero	Frameshift	PV	PVS1,PM2,PP4		0.997	TGS
		c.2492C > T	p.Ala831Val	Hetero	Missense	VUS	PM2,PP4		0.499	
7	7-II-5	c.1669C > T	p.His557Tyr	Homo	Missense	LPV	PM1,PM2,PP3,PP4,PP5	0.991	0.949	TGS
8	8-II-2	c.1669C > T	p.His557Tyr	Homo	Missense	LPV	PM1,PM2,PP3,PP4,PP5	0.991	0.949	TGS
9	9-II-3	c.1604 T > A	p.Ile535Asn	Homo	Missense	LPV	PM1,PM2,PP3,PP4,PP5	0.818	0.949	TGS
10	10-II-3	c.1280G > A	p.Trp427Ter	Hetero	Nonsense	PV	PVS1,PM2,PP4,PP5		0.999	TGS
		c.1604 T > A	p.Ile535Asn	Hetero	Missense	LPV	PM1,PM2,PP3,PP4,PP5	0.818	0.949	
11	11-II-5	c.1604 T > A	p.Ile535Asn	Hetero	Missense	LPV	PM1,PM2,PP3,PP4,PP5	0.818	0.949	TGS
		c.1669C > T	p.His557Tyr	Hetero	Missense	LPV	PM1,PM2,PP3,PP4,PP5	0.991	0.949	
12	12-II-1	c.1669C > T	p.His557Tyr	Homo	Missense	LPV	PM1,PM2,PP3,PP4,PP5	0.991	0.949	WES
13	13-II-5	c.592G > A	p.Gly198Ser	Hetero	Missense	VUS	PM1,PM2,BP4,PP4	0.412	0.675	WES
		c.815G > A	p.Arg272Gln	Hetero	Missense	LPV	PM1,PM2,PP3,PP4	0.741	0.899	
14	14-II-5	c.1669C > T	p.His557Tyr	Homo	Missense	LPV	PM1,PM2,PP3,PP4,PP5	0.991	0.949	WES

*ACMG* American College of Medical Genetics and Genomics; *PV* pathogenic variant; *LPV* likely pathogenic variant; *VUS* variant of unknown significance; *TGS* targeted next-generation sequencing; *WES* whole exome sequencing; *HGVS* human genome variation society; *REVEL* rare exome variant ensemble learner.

Of the variants, c.1669C > T was the most frequently found (9/15 patients, 60.0%). Six of these nine patients (66.7%; 3-III-1, 4-II-3, 7-II-5, 8-II-2, 12-II-1, and 14-II-5) were homozygous for the c.1669C > T variant; their ophthalmologic findings are shown in Fig. 3. The other three patients were heterozygous for the variant, also carrying c.1488del (1-III-1), c.1547T > C (2-III-2), or c.1604T > A (10-II-5). The second most common variant was c.1604T > A; one patient was homozygous for this variant (9-II-3), while another two patients were heterozygous, also carrying c.1280G > A (10-II-3) and c.1669C > T (11-II-5).

### In silico molecular genetic analysis

Among the six identified missense variants, five (all except c.592G > A[p.Gly198Ser]) had a rare exome variant ensemble learner (REVEL) score of above 0.7 and were thus predicted to be pathogenic. For the six variants identified as VUSs according to the guidelines of American College of Medical Genetics and Genomics, the likelihood of pathogenicity was re-evaluated using a Bayesian classifier. With this approach, four VUSs were re-classified as likely pathogenic variants (c.1547T > C, c.1712C > T, c.1604T > A, and c.815G > A) and one likely pathogenic variant was re-classified as a pathogenic variant (c.712del). Only two variants were again classified as VUSs using the Bayesian classifier (c.2492C > T and c.592G > A), with their probability of pathogenicity falling within the range from 0.10 to 0.90.

### Prediction of disease progression

**Figure 4:**
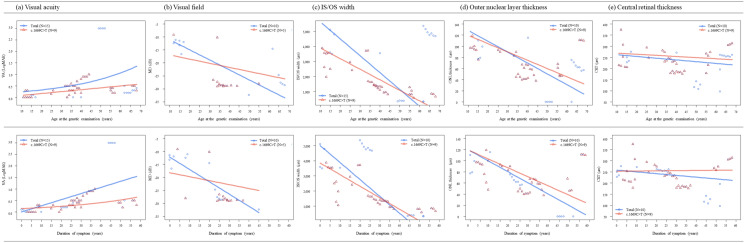
Trends in visual function and retinal morphology with disease progression; analysis based on the application of nonlinear mixed models. (**a**) Deterioration of visual acuity. (**b**) Constriction of the visual field. (**c**) Reduction in the width of the inner segment/outer segment (IS/OS) band. (**d**) Reduction in outer nuclear layer (ONL) thickness. (**e**) Changes in central retinal thickness (CRT). *MD* mean deviation.

Median BCVA was 0.40 LogMAR (range, 0.00–3.00) in the right eye and 0.40 LogMAR (range, 0.00–3.00) in the left eye at the time of genetic testing (Table 1). Based on their worst eye, four patients (4/15, 26.7%) had normal vision (0.10 LogMAR or better); their median age was 27.5 years (range, 14–67 years). Six patients (6/15, 40.0%) showed moderate vision loss (0.1–1.0 LogMAR); their median age was 29.5 years (range, 13–55 years). Five patients (5/15, 33.3%) had poor vision (1.0 LogMAR or worse) and they were relatively older, with a median age of 56.0 years (range, 44–67 years). To evaluate disease progression, changes in VA, VF, and retinal parameters including IS/OS width, CRT, and ONL thickness (ONLT) were analyzed using nonlinear mixed models (Fig. 4 and Table 3). Using data obtained from the longitudinal evaluation of all patients, linear or exponential best fit curves were drawn for age at the time of examination and symptom duration. VA exponentially decreased with age, reaching 1.0 LogMAR or worse at approximately 55 years of age, 35 years after symptom onset. Regarding VF loss, most patients showed moderate to severe loss (− 20 dB ≤ mean deviation <  − 10 dB) early in the second decade of life when central vision was relatively good, and experienced severe VF loss (mean deviation <  − 20 dB) from the age of 35, 20 years after symptom onset. In terms of OCT parameters, the median width of the IS/OS band at the time of genetic testing was 1719.0 μm (range, 328–4715 μm). The IS/OS band width and ONLT decreased linearly at a rate of − 112.5 μm (6.5%)/year and − 2.0 μm (3.2%)/year, respectively, and this was associated with a linear progression of VF constriction seen in most patients. However, CRT was resistant to change during the period of observation. Compared to the total study population, patients with the c.1669c > T variant had a higher VA during follow-up and a lower rate of VF and retinal structure loss.

**Table 3:** The prediction of disease progression in all subjects and in those carrying the c.1669C > T variant, using nonlinear mixed models.

	All subjects							Subjects with the c.1669C > T variant						
	No. of patients	Age at examination, years			Symptom duration, years			No. of patients	Age at examination, years			Symptom duration, years		
		AIC^a^	Beta^b^	*P* value^c^	AIC^a^	Beta^b^	*P* value^c^		AIC^a^	Beta^b^	*P* value^c^	AIC^a^	Beta^b^	*P* value^c^
BCVA, LogMAR^d^	15	− 18.1	0.03	0.001	− 24.4	0.03	< 0.001	9	− 23.7	0.02	0.051	− 25.6	0.03	0.026
VF, MD^e^	10	154.9	− 0.39	0.001	154.7	− 0.47	0.001	5	58.4	− 0.16	0.015	59.1	− 0.15	0.019
IS/OS width, μm^e^	15	882.3	− 112.47	< 0.001	873.2	− 112.47	< 0.001	9	480.6	− 70.43	< 0.001	482.7	− 76.80	< 0.001
ONLT, μm^e^	15	390.4	− 1.96	< 0.001	388.9	− 1.96	< 0.001	9	260.8	− 1.50	0.004	262.6	− 1.59	0.005
CRT, μm^e^	15	493.6	− 0.78	0.281	493.6	− 0.79	0.279	9	310.2	− 0.50	0.566	310.4	0.85	0.924

*BCVA* best-corrected visual acuity; *LogMAR* logarithm of the minimum angle of resolution; *VF* visual field; *MD* mean deviation; *IS/OS* inner segment/outer segment; *ONLT*, outer nuclear layer thickness; *CRT* central retinal thickness; *AIC* akaike information criterion.

For the nonlinear mixed models.

^a^A lower AIC value indicates a better fit.

^b^The beta value represents the estimated rate of disease progression (in units per year) according to age at the time of examination or the number of years since first symptom appearance.

^c^A *P* value was determined for each model.

^d^An exponential growth curve model was used to analyze the changes in BCVA.

^e^While linear growth curve models were used to analyze changes in VF, IS/OS width, ONLT, and CRT.

## Discussion

In this retrospective cohort study, we found that RP patients with *PDE6B* variants exhibited symptoms earlier and were diagnosed earlier than patients with RP caused by other variants. The mean age at diagnosis was 27.5 years (range, 11–60 years) in our cohort. This is similar to that reported in a previous study of RP associated with mutations in the *PDE6* gene family (21.0 years; range, 3–45 years)^12^; however, it is much lower than the mean age at diagnosis reported in a study examining all types of RP in patients of Korean ethnicity (44.8 years; range, 0–95 years)^13^. Considering the age of disease onset, the patients with *PDE6B* variants in our study experienced their first ocular symptoms at a median age of 10.0 years; this is relatively early compared to the median age of onset reported in a previous study of RP associated with other causative genes (29 years; range, 4–77 years)^14^. Similarly, early age of onset of *PDE6B-*RP was observed in a previous study, which found that the average age of onset varied between 10 and 15 years^15^.

In terms of the fundus imaging findings presented in Figs. 2 and 3, the funduscopy and fundus autofluorescence images show that sparse mid-peripheral areas of pigmentation were present in younger patients, while coarse pigmentation invading the macula was observed in older patients; a similar pattern of pigmentary changes with aging was reported in a previous study describing *PDE6B*-RP^16^.

Regarding OCT parameters, ERMs and CME were observed relatively frequently in our patient cohort. An ERM was observed in 53.3% of patients; which was higher than the idiopathic ERM prevalence reported across various populations (1–28.9%)^17^, but within the prevalence range of other studies on RP patients (5–60%)^18^. In our study, ERMs were particularly prevalent in older patients (6/8, 75.0%), likely because aging is a risk factor for ERM development^17^. CME confined to the INL was common in patients with *PDE6B* variants (54.5%); this proportion is higher than that found in a previous study considering CME prevalence across all types of RP (50.9%)^18^. Although the pathogenesis of CME in RP is not fully understood, several hypotheses have been proposed, including Müller cell dysfunction^19^. We suggest that this could be relevant to *PDE6B* mutations because nonfunctional PDE6β subunits lead to an elevated intracellular level of cGMP, resulting in increased Ca^2+^ influx due to decreased channel closure^20,21^. Hence, we can speculate that the increased cation influx causes intracellular K^+^ overload via inward rectifier potassium channels, and this cascade increases the osmotic pressure of Müller cell bodies in the INL, resulting in Müller cell swelling and dysfunction^22^ and ultimately CME and rod cell degeneration^23^.

Among the *PDE6B* variants identified in this study, c.1669C > T was the most common, followed by c.1604T > A. Both variants have been commonly reported in a database of *PDE6B* mutations^24^, but the frequency of variants in our study was notably different to that observed in European patients^12^. Correlating characteristic phenotypes with *PDE6B* variants, we found that CME is frequently found in patients with the c.1669C > T variant. Seven of the nine patients (77.8%) with the c.1669C > T variant had CME; this proportion is notably higher than that observed in RP associated with other variants^18,25^. We postulate that the c.1669C > T variant could account for many cases of RP-related CME, although it is not possible to draw this conclusion based on molecular analysis alone.

Regarding visual function (VA and VF loss), our models suggested that patients carrying the c.1669C > T variant were not expected to have an inferior visual prognosis compared to patients with other *PDE6B* variants (Fig. 4 and Table 3), despite their higher frequency of CME (54.5% in all subjects vs. 77.8% in patients with the c.1669C > T variant). From this finding, we speculate that the existence of CME is not necessarily associated with decreased visual function, as has been found in previous studies^19^. Although photoreceptor disruption has previously been noted in patients with CME affecting multiple retinal layers^26^, CME was confined to the INL in most patients in our study. Therefore, it can be assumed that there was no reason for a radical decrease in ONLT and IS/OS width in patients with CME as opposed to others; thus, the rate of disease progression was not accelerated by the existence of CME. Regarding structural changes, analysis of the IS/OS width and ONLT measured on spectral domain OCT images enabled us to quantify predictions of disease progression. In contrast to previous cross-sectional studies^12^, we created best fit models to correlate anatomical parameters using longitudinal data obtained from long-term follow-up of each patient. Regarding OCT parameters, the median width of the IS/OS band at the time of molecular analysis as 1719.0 μm (range, 328–4715 μm); this value is similar to that reported in a previous study of RP (1914 μm; range, 113–6000 μm)^14^. With respect to disease progression, our nonlinear mixed models revealed that the IS/OS band width decreased linearly at a rate of − 112.4 μm (6.5%) per year; a similar rate was reported in a previous study of RP associated with variants of genes in the *PDE6* gene family, including *PDE6A* and *PDE6B* (− 91 μm [5.9%]/year)^27^. However, the rate observed in this study is higher than that reported in other studies considering all types of RP (reported values vary from − 76.43 μm (4.16%)/year^28^ to − 130 μm (4.9%)/year)^29^ and studies considering autosomal dominant RP (3.8%/year), while it is lower than that reported for X-linked RP (9.4% μm/year)^30^. The generation of sophisticated predictions based on clinical information is a strength of this study.

This single-center, retrospective study has several limitations. First, a cohort of 15 patients is a relatively small number for the analysis of clinical characteristics associated with each variant. Nevertheless, we attempted to minimize the effect of this limitation by using long-term observational data to develop best fit curves for the prediction of disease progression. Secondly, there was a lack of segregation analysis of family data. Only two siblings from one family in this cohort underwent proper genetic analysis; however, co-segregation analysis is important for analyzing *PDE6B* variants, especially VUSs, due to the autosomal recessive pattern of inheritance^31^. Owing to the retrospective nature of this study and a resistance to further study expressed by family members of the subjects, it was not possible to perform further segregation analysis. To address this limitation, we measured the REVEL score for the identified missense variants and conducted further analysis using Bayesian classification for the VUSs. However, future prospective studies are still needed to support the generalization of our findings. Third, the retrospective nature of clinical history collection could have led to recall bias. When analyzing the onset of ocular symptoms or the date of diagnosis at external clinics, we had to rely on patients’ reports in their medical records, and this could potentially affect the accuracy of our predictions of visual prognosis. However, we were able to use long-term clinical data to quantify the rate of disease progression and we found that our results are in good agreement with recent studies evaluating the rate of disease progression. Therefore, we believe that this study effectively uses genotype–phenotype matching to predict visual function and retinal structure loss in patients with *PDE6B* variants.

In conclusion, this study described 11 *PDE6B* variants identified by TGS and WES in patients with autosomal recessive RP, and used long-term clinical data to quantify the rate of disease progression based on OCT findings and visual prognosis. Our data also enable us to comment on genotype–phenotype correlations, especially for the c.1669C > T variant. This study highlights the potential of next-generation sequencing data for exploring the relationships between disease associated gene variants and phenotypes.

## Methods

### Patients

This retrospective cohort study was conducted at a single tertiary clinic, the Asan Medical Center (Seoul, Korea). Records of 305 patients clinically diagnosed with RP who underwent molecular diagnosis between March 2018 and December 2019 were reviewed, and the patients’ pathogenic variants were screened. A total of 15 patients from 14 families with *PDE6B* variants were recruited and their ophthalmologic clinical data were obtained from medical records, including comprehensive information regarding clinical and familial history and the first ocular symptoms of the disease. The study protocol adhered to the tenets of the Declaration of Helsinki and the study design was approved by the institutional review board of the Asan Medical Center. The requirement for written informed consent was waived by the institutional review board of the Asan Medical Center (IRB no. 2019-0182) due to the retrospective nature of this study.

### Ocular examinations

All patients underwent detailed ophthalmologic examinations through dilated pupils including measurement of BCVA, slit-lamp biomicroscopy, funduscopic examinations, fundus photography, fundus autofluorescence imaging (Optos, Dunfermline, UK), and full-field electroretinography (Roland-Consult, Brandenburg, Germany) to confirm the diagnosis of RP, according to the standards of the International Society for Clinical Electrophysiology of Vision. Spectral domain OCT (Heidelberg, Dossenheim, Germany) images were examined to evaluate the retinal structures. An ERM was considered to be present if hyperreflectivity was observed above the ILM surface with or without foveal distortion, whereas CME was diagnosed if hyporeflective cystic spaces were present on one or more consecutive raster scans crossing the fovea. The width of the IS/OS band, CRT, and ONLT for every year of follow-up were measured on horizontal macular scans using the built-in measurement scale provided in the spectral domain OCT software; this enabled evaluation of the rate of IS/OS constriction and ONL thinning with disease progression. For serial evaluations, patients whose IS/OS band and ONL could not be detected on OCT images for at least two years were excluded. Also, the static Humphrey visual field test (HFA 750I, Carl Zeiss Meditec, Dublin, CA, USA) and Goldmann kinetic perimetry (Haag-streit AG, Köniz, Switzerland) were performed to evaluate patterns of VF defects. All clinical data and the accuracy of diagnosis were confirmed by two retinal specialists (YN Kim, JY Lee).

### Analysis of genetic variants

Genomic DNA was extracted from peripheral blood samples taken from patients. TGS was performed for 10 of the 15 patients with the Ion Torrent S5XL platform (Thermo Fisher Scientific Inc., Waltham, MA, USA) using a panel consisting of 88 genes associated with RP, and for further two patients with the Illumina NextSeq platform (Illumina Inc., San Diego, CA, USA) using the TruSight One Sequencing Panel. For the remaining three patients, WES was performed. All exons of all genes (approximately 22,000) were captured using a SureSelect kit (Version C2; Agilent Technologies Inc., Santa Clara, CA, USA). The captured genomic regions were sequenced using a NovaSeq platform (Illumina Inc., San Diego, CA, USA). Analysis of raw genome sequencing data included alignment to the reference sequence (NCBI genome assembly GRCh37; accessed in February 2009). For TGS, mean depth of coverage was approximately 500-fold, with 99.2% coverage higher than 20-fold. Variant calling, annotation, and prioritization were performed as previously described^32^. The requirement for verification of identified variants was waived for Torrent S5XL sequencing data when the read depth was over 100 reads and the allele frequency was 40–60%^33^, while validation of Illumina NextSeq and WES data was achieved with subsequent Sanger sequencing.

### In silico molecular genetic analysis

All identified variants were classified according to the guidelines of the American College of Medical Genetics and Genomics^11^. To evaluate the predicted functional effect and the degree of evolutionary conservation of identified variants, the REVEL method was applied among several in silico tools. REVEL is an ensemble method that incorporates 18 individual scores from 13 different tools (MutPred, FATHMM, VEST, PolyPhen, SIFT, PROVEAN, MutationAssessor, MutationTaster, LRT, GERP, SiPhy, phyloP, and phastCons) to produce a pathogenicity score for missense variants; in a previous study, this method showed the best performance compared to seven other ensemble methods (MetaSVM, MetaLR, KGGSeq, Condel, CADD, DANN, and Eigen)^34^. The missense variants with a REVEL score of above 0.7 were predicted to have a detrimental effect^34,35^. The pathogenicity of variants identified as VUSs was re-evaluated by calculating their posterior probabilities obtained using a Bayesian calculator^36^. Variants were classified as pathogenic if the probability was over 0.99; the likely pathogenic threshold was 0.90, and variants with a probability of 0.10 to 0.90 were considered to be VUSs.

### Statistical analysis

The eye studied was randomly selected for each patient. Descriptive statistics (number and percentage for categorical variables; mean and median for continuous variables) were calculated to summarize the baseline characteristics of patients. Statistical analyses for these characteristics were performed using SPSS Statistics for Windows, version 21 (IBM Corp., NY, USA). To evaluate how visual acuity (VA; LogMAR) and OCT parameters (IS/OS width, CRT, and ONLT) varied according to age at the time of examination and number of years since first symptom appearance, nonlinear mixed models^37^ were developed using SAS version 9.4 (SAS Institute, Inc., NC, USA).

Linear and exponential growth models were compared and the model with the smaller Akaike Information Criterion was selected as the best fit. A *P* value of < 0.05 was considered statistically significant.

An exponential growth curve model was used to analyze the changes in BCVA:

$$ {\text{y }} = {\text{ a}} \cdot {\exp}\left( {{\text{beta}} \cdot {\text{t}}} \right) $$

For the analysis of VF, IS/OS width, ONLT, and CRT, a linear growth curve model was used:

$$ {\text{y }} = {\text{ a }} + {\text{ beta}} \cdot {\text{t}} $$

## Data Availability

The datasets generated during and/or analyzed during the current study are available from the corresponding author on reasonable request.
